# The BetterBirth Program: Pursuing Effective Adoption and Sustained Use of the WHO Safe Childbirth Checklist Through Coaching-Based Implementation in Uttar Pradesh, India

**DOI:** 10.9745/GHSP-D-16-00411

**Published:** 2017-06-27

**Authors:** Nabihah Kara, Rebecca Firestone, Tapan Kalita, Atul A Gawande, Vishwajeet Kumar, Bhala Kodkany, Rajiv Saurastri, Vinay Pratap Singh, Pinki Maji, Ami Karlage, Lisa R Hirschhorn, Katherine EA Semrau

**Affiliations:** a Ariadne Labs, a Joint Center between Brigham and Women's Hospital and the Harvard T.H. Chan School of Public Health, Boston, MA, USA.; b Population Services International, Washington, DC, USA.; c Population Services International, Lucknow, Uttar Pradesh, India.; d Ariadne Labs, Boston, MA, USA; Department of Health Policy and Management, Harvard T.H. Chan School of Public Health, Boston, MA, USA; Department of Surgery, Brigham and Women's Hospital, Boston, MA, USA.; e Community Empowerment Lab, Lucknow, Uttar Pradesh, India.; f Jawaharlal Nehru Medical College, Belgaum, Karnataka, India.; g Ariadne Labs, Boston, MA, USA; Feinberg School of Medicine, Northwestern University, Chicago, IL, USA.; h Ariadne Labs, Boston, MA, USA; Department of Medicine, Harvard Medical School, Boston, MA, USA; Division of Global Health Equity, Department of Medicine, Brigham and Women's Hospital, Boston, MA, USA.

## Abstract

Shifting childbirth into facilities has not improved health outcomes for mothers and newborns as significantly as hoped. Improving the quality and safety of care provided during facility-based childbirth requires helping providers to adhere to essential birth practices—evidence-based behaviors that reduce harm to and save lives of mothers and newborns. To achieve this goal, we developed the BetterBirth Program, which we tested in a matched-pair, cluster-randomized controlled trial in Uttar Pradesh, India. The goal of this intervention was to improve adoption and sustained use of the World Health Organization Safe Childbirth Checklist (SCC), an organized collection of 28 essential birth practices that are known to improve the quality of facility-based childbirth care. Here, we describe the BetterBirth Program in detail, including its 4 main features: implementation tools, an implementation strategy of coaching, an implementation pathway (Engage-Launch-Support), and a sustainability plan. This coaching-based implementation of the SCC motivates and empowers care providers to identify, understand, and resolve the barriers they face in using the SCC with the resources already available. We describe important lessons learned from our experience with the BetterBirth Program as it was tested in the BetterBirth Trial. For example, the emphasis on relationship building and respect led to trust between coaches and birth attendants and helped influence change. In addition, the cloud-based data collection and feedback system proved a valuable asset in the coaching process. More research on coaching-based interventions is required to refine our understanding of what works best to improve quality and safety of care in various settings. (After publication of this article, the impact results of the BetterBirth intervention were published in the New England Journal of Medicine [volume 377, pages 2313-2324, doi: 10.1056/NEJMoa1701075]. The results showed that the intervention had no significant effect on maternal or perinatal mortality or maternal morbidity, despite having positive effects on essential birth practices.)

## Abstract (precis)

The BetterBirth Program relied on carefully structured coaching that was multilevel, collaborative, and provider-centered to motivate birth attendants to use the WHO Safe Childbirth Checklist and improve adherence to essential birth practices. It was scaled to 60 sites as part of a randomized controlled trial in Uttar Pradesh, India.

See related article by Marx Delaney.

## INTRODUCTION

The reduction of preventable maternal and neonatal morbidity and mortality associated with childbirth remains a critical challenge in global health.^1^^,^^2^ Previously, countries—especially low- and middle-income countries—have embraced interventions focused on encouraging childbirth to take place in health care facilities; however, despite the success of many of these interventions, the shift to facility-based childbirth has not succeeded in improving all childbirth-related outcomes at the levels expected.^3^ Improving the quality of care received by mothers and newborns during facility-based childbirth is the next step in improving these health outcomes.

One important component of high-quality health care is the provision of care according to evidence-based guidelines. In facility-based childbirth care, one of the main causes of preventable harm is the failure to deliver essential birth practices to all mothers and newborns at the appropriate time during childbirth. Essential birth practices are provider behaviors for which evidence exists to prove that they increase the quality and safety of care during childbirth; these practices, when performed consistently and correctly, can save the lives of mothers and newborns. The failure to perform these practices is often called a “know-do” gap and has been identified in many areas of health care.^4^

One of the main causes of preventable harm in facility-based childbirth care is the failure to deliver essential birth practices.

In specific health care settings, well-implemented checklists have successfully bridged the know-do gap, changing provider behavior by increasing adherence to evidence-based guidelines.^5^ Studies have demonstrated that this approach to improving quality of care has significantly improved outcomes in intensive care medicine and in surgery, including in resource-limited settings.^6^^,^^7^ Based on these successes, the World Health Organization (WHO) and collaborators developed the WHO Safe Childbirth Checklist (SCC), a collection of 28 evidence-based essential birth practices associated with improved maternal and neonatal outcomes.^8^^,^^9^

Experience with the WHO Surgical Safety Checklist and with similar quality-improvement and patient-safety interventions has established that simply introducing a checklist to a facility or provider without a plan for engagement and sustained reinforcement does not lead to improvement in health care practices.^10^^–^^13^ Ensuring consistent adherence to these practices requires both their codification into guidelines, as well as deliberate behavior change interventions to support adoption of these evidence-based practices.^14^

While checklists have successfully bridged the know-do gap, experience has shown that a plan for engagement and sustained reinforcement is also needed to improve health care practices.

With the BetterBirth Program, we aimed to develop a systematic approach that would enable health care workers to adopt and use the WHO Safe Childbirth Checklist during their provision of childbirth care. As such, we sought to empower birth attendants and other facility and district personnel to identify, understand, and ultimately resolve barriers they might face in using the SCC to deliver quality maternal and newborn care, with coaching as the main strategy to engage with health care workers. The BetterBirth Trial, a matched-pair, cluster-randomized controlled trial, was implemented in 120 facilities (60 intervention, 60 control) across 24 districts in the state of Uttar Pradesh, India.^15^ Evidence of the impact of the BetterBirth Program on birth attendants' performance of essential birth practices and on health outcomes for mothers and newborns is forthcoming.

Behavior change interventions and adoption of evidence-based practices are enhanced by application of a theoretical framework to organize strategies of behavior change.^14^ To develop the BetterBirth Program, we modified the Opportunity-Ability-Motivation (OAM) framework of behavior change to ground our strategy design, particularly in how we aimed to structure coaching and supervision of health care workers.^14^^,^^16^^–^^18^ Originally, the OAM framework was used to describe consumer behavior; to make it more applicable to our work—given the prevalence of supply-related challenges in resource-limited settings—we separated “Supply” from the more general category of “Opportunity.” Thus, our modified OAMS framework contained 4 categories into which barriers to behavior change can be classified:

- **Opportunity:** “external” and systems-level barriers concerning the circumstances under which the provider must practice the new behavior (e.g., the amount of staff available, the time available for performing the practice, the size of the facility/number of beds available, the characteristics of the patient population)
- **Ability:** barriers concerning the provider's knowledge, skills, and competence related to the new behavior (e.g., clinical skills, communication skills)
- **Motivation:** “internal” barriers concerning the provider's willingness to change his or her behavior (e.g., understanding and believing in the significance of the new behavior)
- **Supplies:** a subset of Opportunity, specifically referring to the availability of necessary medications, equipment, and other consumable materials

Here, we describe the design of the BetterBirth Program in detail. We include specific, concrete examples to illustrate the various aspects of our intervention: quotes that the implementation team gathered in focus group discussions with coaches and other examples drawn from the Coach Support Tools (qualitative data collection tools used by coaches during their work in facilities). We have described the methodology of the BetterBirth Trial,^15^ including pilot testing of the intervention,^19^ elsewhere. A companion article published in this issue of *Global Health: Science and Practice* presents results on the effect of the intervention on birth practices.

## INTERVENTION: THE BETTERBIRTH PROGRAM

The BetterBirth Program incorporated 4 key components: implementation tools, an implementation strategy, an implementation pathway, and a sustainability plan.

### BetterBirth Implementation Tools: SCC and Pulse

By reminding birth attendants to perform essential birth practices at the appropriate time, the SCC is intended to improve the safety and quality of care received by mothers and newborns. The SCC is organized into 4 pause points, or critical moments, in facility-based childbirth care, when birth attendants should “check” that they have completed essential birth practices: (1) on admission, (2) just before pushing (or before cesarean delivery), (3) soon after birth (within 1 hour), and (4) at discharge (Supplement). These pause points allow birth attendants to make their “checks” both at critical times when they can protect the mother and newborn against dangerous complications and when it is convenient for them to take the time to perform the checks.^9^

The WHO Safe Childbirth Checklist is organized into 4 pause points when birth attendants should check that they have completed essential birth practices: on admission, just before pushing, soon after birth, and at discharge.

In the BetterBirth Trial, birth attendants completed a new paper version of the SCC—which was initially adapted to fit the relevant national guidelines of India—for each mother and attached it to the mother's chart or bedhead ticket to allow them to more easily track practices for each mother-and-baby pair.^15^ Most commonly, the birth attendants applied the “Read-Do” method: they first read the item on the SCC, then completed the task. Alternatively, they used the “Do-Confirm” method, in which they completed the task then immediately read the item on the paper or poster SCC to confirm that it was performed appropriately. As they completed each essential birth practice, birth attendants put a check in the box located to the left of the item to indicate completion. Relevant notes, such as temperature and blood pressure readings, were written to the right of each item, where additional information is provided to guide the birth attendant in clinical decision making. For example, prompts to check temperature and other clinical signs are noted to the right of the SCC item related to the administration of antibiotics. While the SCC was primarily completed by an individual birth attendant for each patient, it was sometimes used as a team, particularly in the case of organizing supplies for mother and baby as indicated in Pause Point 2.

In a pilot study, with successful adoption and sustained use of the SCC, birth attendants demonstrated improved adherence to essential birth practices.^19^^,^^20^ Moreover, the SCC further facilitated implementation of the BetterBirth Program by serving as a tool for coaches. The organized list of essential birth practices on the SCC served as the foundation of coaches' observations of birth attendants' behavior.

**FIGURE 1:**
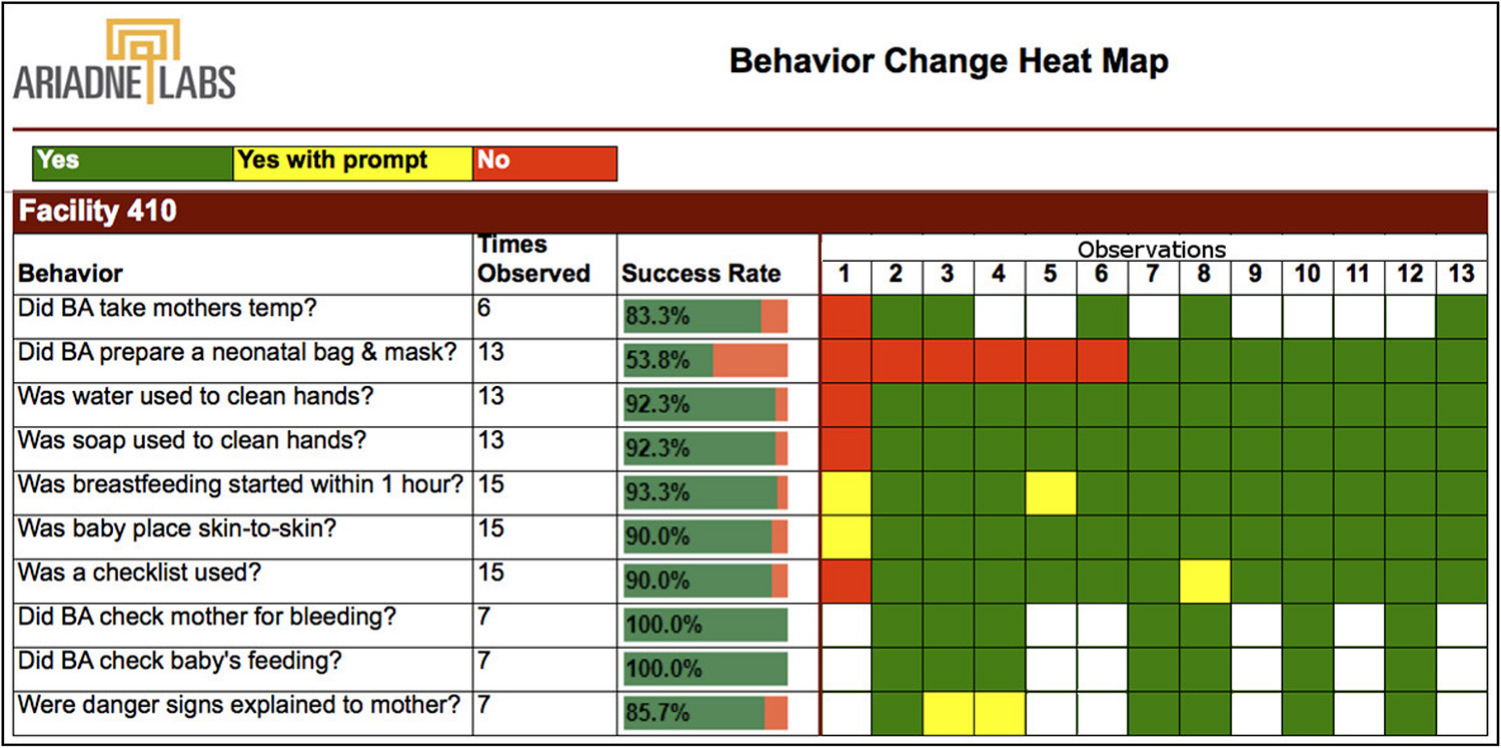
Partial Facility-Level Heat Map of Adherence to Essential Birth Practices, Illustrative Example (Not Real Data) Success rates calculated as follows: green=1, yellow=0.5, red=0, white=N/A. Abbreviation: BA, birth attendant.

Built to facilitate the implementation of the BetterBirth Program and the management of the BetterBirth Trial, Pulse is a management information system designed to provide rapid feedback on implementation status. Operable on mobile phones and tablets, Pulse provided near-real-time access to information about the use of the SCC, adherence to specific essential birth practices, and supply availability. A data entry app developed by Dimagi (http://www.dimagi.com/products/), called CommCare, allowed coaches to record their observations and put those observations to use in further coaching. For example, a coach would record whether a birth attendant performed specific practices (such as skin-to-skin warming and breastfeeding), and if not, diagnose and record the underlying barrier according to the OAMS framework. Coaches and coach team leaders were able to access a summary report of that birth attendant's practices or an aggregate report of the facility's practices. These summary data were generally displayed in a visual heat map (Figure 1), allowing coaches, birth attendants, and other facility and district personnel to more easily recognize trends in the performance of essential birth practices and underlying barriers preventing behavior change. Finally, Pulse facilitated the overall implementation of the BetterBirth Program by aggregating coaches' observations at the facility and district levels to help various stakeholders analyze systemic trends and barriers to SCC use.

Visual heat maps allowed coaches and birth attendants to more easily recognize trends in the performance of essential birth practices.

### BetterBirth Implementation Strategy: Coaching

We understood from previous work with checklists and other quality-improvement and patient-safety programs that changing behavior is a challenging process requiring ongoing support.^21^^–^^23^ Thus, we looked to other fields' successes for inspiration. In sports,^24^ business,^25^ and education,^26^ coaching has been a popular and successful strategy for changing individuals' performance. One such example of a successful behavior-change intervention is the U.S. agricultural extension service, which was established in 1911 to help make American farmers' practices more modern and efficient by incorporating then-new scientific techniques (Box). Client-centered, collaborative, multilevel, in-person support—or coaching—allowed the Green Revolution to flourish.^27^ These crucial lessons, as well as lessons from other models such as the Improvement Collaborative Approach, provided a foundation in designing the BetterBirth coaching strategy.^28^ BetterBirth coaching emphasized the individual barriers to adopting essential birth practices and also employed a team-level approach to address systemic barriers to adoption.

BOX **Lessons Learned About Behavior Change From the U.S. Agricultural Extension Service**

The success of the U.S. agricultural extension service resulted from how it pursued its goals: extension agents went out “into the field” to work with local farmers on integrating new developments in technology and farming techniques into the farmers' work. Moreover, each extension agent was guided by certain principles, which led to more successful relationships with the farmers:

- **Farmer-centered:** the agents paid close attention to and responded directly to the farmers' needs, and the agents' work was guided directly by a council comprised of local farm-community leaders.
- **Collaborative:** the agents invited the farmers to participate extensively in the processes of identifying what the local community needed, planning solutions, and evaluating the success of those programs.
- **Multilevel:** the agents also worked closely with other levels of the agricultural research establishment (such as the agronomy research departments at the then-young land-grant universities).

In the program, nurse coaches worked closely with birth attendants, who were also typically nurses, to provide the support that the birth attendants needed in order to use the SCC to improve care. Simultaneously, coach team leaders, who were either physicians or public health professionals, supported facility and district leaders as they strengthened the health care systems' facilitation of the use of the SCC. To ensure a local facility-level champion, coaches and facility leaders collaboratively chose and provided support to a childbirth quality coordinator (CQC) (an individual or a small team), who conducted many of the coaching tasks in the absence of the BetterBirth coach. The CQC was identified during the first 2 months of the program. Please see the Table for details on qualifications and selection criteria for each of these roles. Within the BetterBirth Trial, coaching entailed 3 main tasks and was characterized by 3 main principles, enumerated below.

**TABLE.** Selection Criteria and Responsibilities of BetterBirth Program Team Members

Team Member	Qualifications/Selection Criteria	Responsibilities
Coach	Nurse qualification Trained in childbirth practices Recruited from same hub as facility assignments	Coach facility birth attendants (increase motivation, observe, and facilitate problem solving) Manage 2–4 facilities at any one time; conduct 43 visits per facility
Coach Team Leader	Physician or trained public health practitioner At least 4 years of experience Recruited from same hub as facility assignments	Provide supportive supervision to the coach Coach facility and district leaders to strengthen the health care system Manage 4–5 facilities at one time; conduct 23 visits per facility
Childbirth Quality Coordinator	Facility-based staff Motivated and interested in the BetterBirth Program Ability to influence and coach others Well respected among other facility- and district-level personnel	Orient new staff to the Safe Childbirth Checklist Coach facility birth attendants Coach facility and district leaders to strengthen the health care system Collect data on facility progress and areas for improvement

To prepare coaches, each coach attended a 5-day interactive training that focused on core coaching skills, such as effective communication, respectful relationship building, and barrier and solution identification, as well as on the OAMS behavior-change framework. Throughout the trial, coaches and coach team leaders also received 2–3 day refresher trainings, which offered similar skills training and opportunities for peer learning and troubleshooting. Coach team leaders also provided supportive supervision to coaches during facility visits, in which both coaches and coach team leaders were present.

#### Three Coaching Tasks

Coaches pursued 3 main types of activities during their facility visits. First, based on local circumstances and dynamics, coaches worked to *increase birth attendants' motivation* to use the SCC. If a birth attendant did not perform a specific essential birth practice, a coach might explain the significance of that practice by using the SCC as a visual aid or by telling a story about a childbirth in which the practice was performed. Coaches had to identify what motivated each birth attendant—competition among peers or other facilities, facts and data, or emotional stories—and tailor their approach accordingly. Coaches would keenly observe the birth attendant and the contextual environment, as well as probe with open-ended questions, to understand the root cause of the barrier. When describing how coaches overcame resistance to using the SCC, one coach noted:

We have to empathize with the birth attendant and explain the importance of conducting each item on the SCC. As humans, it's not always possible for us to remember each item—especially when we are busy.

Similarly, coaches celebrated success, both with the birth attendant and among the birth attendant's peers within the facility.

Coaches pursued 3 main types of activities: motivated birth attendants to use the checklist; observed, recorded, and fed back information about use of the checklist; and guided birth attendants to solve problems.

Second, using the Pulse app, coaches *observed, recorded, and fed back information* about SCC use, the status of the facility's systems (such as supply availability), and any barriers the birth attendant, facility, and/or district faced in using the SCC. By making birth attendants and facility leaders aware of the performance (and nonperformance) of essential birth practices within the facility, the coaches helped to identify areas for improvement. The importance of this task draws from the lessons learned with the Standards-Based Management and Recognition (SBM-R) model.^29^ Ongoing measurement of performance was recorded to guide the improvement process and motivate the providers to improve care over time. Coaches and coach team leaders recorded their observations in Pulse using a quantitative tool, which also captured the specific barrier to performing the practice using the OAMS framework. The reports generated from Pulse showcased activities performed without prompting in green, while practices that were prompted were marked yellow, and practices not performed at all were marked red (Figure 1). For each observation marked red (not performed), a barrier was also indicated to describe why: O for opportunity, A for ability, M for motivation, and S for supplies. Coaches remarked that birth attendants were motivated by the heat maps and worked to turn “reds” (unperformed practices) into “greens” (practices performed unprompted).

Similarly, Coach team leaders collected information on and offered feedback about the state of a facility's systems and practices to facility leaders. When coaches were not able to directly observe any of the pause points during the facility visit, they still found opportunities to discuss past cases, current supply gaps, and skilled birth attendant national guidelines. Coaches and coach team leaders also completed structured diaries for each facility visit and district engagement that captured qualitative information about each of their interactions, drawing not only from direct observations but also from discussions related to practices and processes even in the absence of direct observations. The coach team leader qualitative tool specifically captured information on team-based coaching discussions and data feedback meetings, particularly around supply availability as well as facility-level practice and processes changes.

In the third task, *problem solving*, coaches guided birth attendants through a problem-solving process based on the results of the second task (observe, record, and provide feedback). When the coach identified an essential birth practice that the birth attendant did not perform even after prompting from the coach, the coach collaborated with the birth attendant to identify what barrier blocked her from performing the practice and to categorize that barrier according to the OAMS framework. Finally, the coach and the birth attendant agreed upon a strategy for resolving the identified barrier and worked together as a team toward realizing that strategy. The coach team leader helped by facilitating the escalation of the problem to higher levels of facility and district leadership if needed, where the coach team leader also facilitated the problem-solving process. When there was turnover of staff and shift changes, coaches and coach team leaders would make every effort to arrange their visits such that they could work individually with as many birth attendants at the facility.

Coaches did not typically have problems finding time with birth attendants to discuss cases and review feedback and thus were generally able to build a strong relationship and rapport with each birth attendant. When coaches observed that birth attendants were routinely not performing the tasks associated with the fourth pause point (at discharge), they engaged the birth attendants in discussions to determine what barriers were preventing the adherence to these essential birth practices. The birth attendants pointed out that, for cultural and logistical reasons (e.g., wanting to introduce the new baby to family members, needing to return home to care for other children, not having access to food in the facility), mothers often left the facility—against medical advice—much earlier than the expected 24 hours postpartum recommended by the SCC. Their early departures left no time for the birth attendants to perform the fourth pause point. Together, birth attendants and coaches agreed to administer the discharge practices (pause point 4) regardless of how soon the mother left the facility. They also decided together to approach the female community health workers (Accredited Social Health Activists or ASHAs) who accompanied women to the facility for childbirth in order to educate them on the need for women to remain in the facility for 24 hours after giving birth. The birth attendants shared with the ASHAs the potential dangers of leaving the facility early and warning signs to watch for once a mother had returned home. As a result, the ASHAs began to encourage mothers to remain at the facility longer post-delivery.

#### Three Coaching Principles

*What* coaches focused on was only one part of the intervention. Equally, if not more, important was *how* the coaches pursued their tasks. First, coaching in the BetterBirth Program was *multilevel*. While coaches worked with birth attendants, coach team leaders similarly worked with facility and district leaders to strengthen the systems necessary to facilitate sustained use of the SCC. For example, coaches noticed birth attendants refusing to administer the bacille Calmette-Guérin (BCG) vaccine to only 1 or 2 babies at a time, instead asking the mothers to return to the facility at a later time for the vaccine. Discussions with birth attendants revealed that they believed they were required to use all 10 doses of vaccine in each vial immediately upon opening it; thus, to avoid waste, they would only vaccinate if many babies were present in the facility at once. To resolve the misunderstanding, coaches and coach team leaders worked with facility- and district-level health care leaders to clarify the policy around the supply of BCG vaccine. In addition, at a district-level meeting, leaders explained to the birth attendants the ready availability of plenty of vaccine as well as the procedures for acquiring more for their facility, in order to motivate them to vaccinate each baby—to open a vial even when only 1 baby was present. To be successful, this effort required collaboration not only between the peer-to-peer pairings of coaches and birth attendants, coach team leaders, and health care leadership but also between the 2 pairs as well.

Coaching was multilevel, collaborative, and provider-centered.

Second, coaching in the BetterBirth Program was *collaborative*. Coaches worked with birth attendants, and coach team leaders worked with facility and district leaders, in a supportive, constructive, respectful peer-to-peer manner. The coaches were also nurses (and, due to cultural norms, female) with similar backgrounds and training as the birth attendants they coached; the coach team leaders who worked with facility and district leaders—both clinical and administrative—were physicians or experienced public health professionals. Coaches and coach team leaders used strong communication skills and a nonjudgmental attitude to build a relationship of trust and understanding with the individual(s) they coached. One birth attendant remarked:

At first I didn't think that this young BetterBirth Coach could help me very much. But she was very polite and pleasant to work with. The coach helps me to remember and learn to do each practice in a systematic and consistent way for every patient. I bring the skills, she brings the process.

Another noted:

It wasn't like talking to someone who was trying to find mistakes. It was like talking to a friend.

Birth attendants and facility and district leaders thus were invited to be active, equal participants in the process of improvement.

Third, coaching was *provider-centered*. Similar to the Client-Oriented, Provider-Efficient services model (COPE), coaching focused on enabling providers to identify problems and develop their own solutions using local resources.^30^ At all levels of the health care system, coaches set local priorities for the implementation of the BetterBirth Program based not upon the expectations and needs of the coaches and the coach team leaders but upon the expectations and needs of the birth attendants and facility and district leaders they coached. The initial priorities that coaches and coach team leaders addressed in each facility, for example, were determined by the facility personnel at the launch events; these ranged from clarifying referral protocols to handling biological waste to improving communications. As a result of this provider-centered attitude, coaches needed to be nimble and adaptive to differing circumstances and contexts as the priorities of birth attendants and facility and district leaders shifted. Overall, coaching in the BetterBirth Program was customized to the specific individual being coached and the situation in which the individuals were working.

### BetterBirth Implementation Pathway: Engage, Launch, Support

**FIGURE 2:**
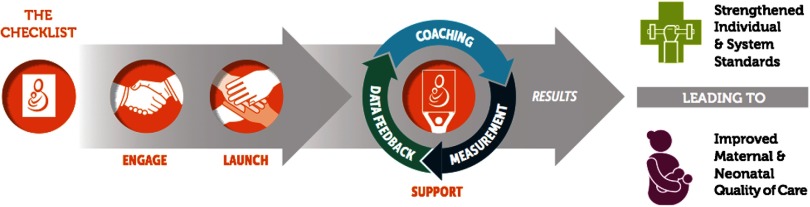
BetterBirth Implementation Pathway, Uttar Pradesh, India

The BetterBirth implementation pathway incorporated 3 stages: Engage, Launch, and Support (Figure 2). Each stage involved discrete, sequential goals that built upon one another. Together, the 3 stages linked together in a pathway leading from the first collaboration between implementers and key stakeholders to a process aimed at improved safety and quality of care for mothers and newborns during facility-based childbirth care.

The core of the *Engage* stage of the BetterBirth implementation pathway was collaborating with key leaders at multiple levels of the health care system to introduce them to the SCC and gather support for the BetterBirth Program, identify local needs, and engage in problem solving to begin addressing those needs.

During the Engage stage of the BetterBirth Trial in Uttar Pradesh, India, implementers worked closely with state health care officials to adapt the SCC to fit the Government of India maternal and child health guidelines. Coach team leaders from the BetterBirth Program also met with facility and district leaders in order to secure their commitment to the BetterBirth Program and to identify their priority areas for improvement.

The *Launch* stage aimed for collaboration between BetterBirth Coaches and those individuals who would be adopting and using the SCC (and those who would be directly supporting them). This stage involved introducing the key stakeholders to the SCC and gathering support for the BetterBirth Program's goals and methods, beginning to identify local barriers to adopting essential birth practices, and engaging in problem solving to begin resolving those barriers. BetterBirth Coaches led a “launch event” with facility personnel, seeking both to strengthen the sense of responsibility and motivation for ensuring high-quality care in childbirth services and to create an atmosphere of excitement and inspiration that would build confidence in the idea of behavior and system change.

During the Launch stage in the BetterBirth Trial, coaches used a flipbook illustrated with diagrams of SCC essential birth practices and motivational multimedia presentations to engage participants in a discussion about why each practice on the SCC is critical for a safe childbirth.^31^^–^^33^ Coaches also facilitated a gap analysis, in which each facility's personnel—including those not directly involved in providing care during childbirth (e.g., pharmacists)—began the process of identifying the barriers to using the SCC in their facility and brainstorming resolutions for those barriers. These participatory gap-analysis discussions also served to guide coaches and coach team leaders in choosing initial areas of focus.

The central goal of the *Support* stage was to bolster the adoption and ongoing use of the SCC within facilities and to reinforce birth attendants' adherence to essential birth practices. To this end, coaches and coach team leaders visited facilities and worked directly with birth attendants and facility leaders (as well as with other facility personnel) to increase motivation, collect data, provide feedback, and problem solve around barriers to behavior change.

The Support stage of the BetterBirth Trial in Uttar Pradesh involved coaches visiting each intervention facility 43 times over 8 months. During the first 4 months, coaches visited each facility twice per week, then the frequency of their visits tapered until they visited each facility only once in the eighth month. Coach team leaders followed a similar but less intensive schedule, visiting each facility 23 times. Each coach was responsible for 2–4 facilities at a time; each coach team leader managed 4–5 facilities at a time.

Coaches visited each intervention facility 43 times over an 8-month period.

### BetterBirth Sustainability Plan: Coaching for Empowerment and the Childbirth Quality Coordinator

BetterBirth Coaches and Coach Team Leaders provided neither monetary nor other material benefits to the individuals or facilities they coached, nor did they offer those individuals direct skills training. Instead, a coach's central goal was to *empower* health facility leadership and staff. Coaching helped birth attendants and facility and district leaders to recognize the barriers they faced in using the SCC, and helped them develop the strategies to resolve those barriers by using the resources already available within the facility and the district—and, when possible, by strengthening the facility and district systems to support the use of the SCC. By empowering the birth attendants and facility and district leaders through coaching, the BetterBirth Program sought to create culture and capacity change that would last beyond the coaches' visits. As one birth attendant commented:

My coach helps to show me the path to where the solution exists. Now I feel confident to bring up issues to the MOIC [Medical Officer In Charge] or LMO [Lady Medical Officer].

During the Support stage, coaches also collaborated with the personnel in each facility in order to produce a local sustainability plan for continuing to pursue the goals, methods, and effects of the program after the coaches moved on to other facilities. The local sustainability plan offered facility personnel another tool with which they could continue to practice and advocate better safety and quality of facility-based childbirth care.

As a second part of the sustainability plan, a CQC was collaboratively chosen early in the BetterBirth Program by coaches and facility leaders to be the “local champion” of the SCC. The choice of facility CQC was based on a staff member's motivation and interest in the BetterBirth Program, his or her ability to influence and coach others, and the level of respect she or he held among other facility- and district-level personnel, rather than on his or her title or role in the facility. With support from coaches and facility leaders, the CQC was responsible for the use of the SCC within the facility during and after the implementation of the BetterBirth Program, making certain that the facility practiced the principles embodied by the BetterBirth Coaches, even when those coaches were not present. According to one CQC:

We gained energy to continue quality and infection control measures and keep on improving [the] BetterBirth Program.

Once the CQC was identified during the first 2 months of coaching, coaches oriented and supported CQCs in basic coaching skills for approximately 6 months before the intervention period ended. Within the intervention period, a formal training session was also organized at the district level to train the facility and district CQCs in observation and data collection techniques, delivering feedback, and other coaching methods. Practically, the CQC oriented new staff to the SCC, and continually motivated all staff in its adoption and use, and monitored and aided staff members in collaboratively addressing facility- and district-level barriers to SCC adherence. Another CQC reported:

… a very high motivation and self-confidence that safe birth practices can be very well implemented and continued even in the absence of BetterBirth. I can manage and check things and supplies very easily.

The CQC received no extra incentives for playing this role within the facility or district.

## LESSONS LEARNED

Although the BetterBirth Program revolved around using the WHO Safe Childbirth Checklist to improve essential birth practices, coaching was at the core of the program. Recognizing that checklists alone do not resolve all the barriers to behavior change, we adapted lessons from other behavior-change strategies, including the Improvement Collaborative Approach, COPE, and SBM-R,^28^^–^^30^ into a public health intervention responsive to local needs. Further research is still required to understand what components of coaching, both individual and team-based, are most effective in influencing behavior uptake and other systemic change, and in which contexts. The BetterBirth Program required performing the coaching tasks (increasing motivation, measuring and offering feedback, problem solving) and operationalizing the coaching principles (multilevel, collaborative, provider-centered) in order to achieve adoption and sustained use of the SCC. BetterBirth Coaches' emphasis on relationship building and respectful communication during measurement-and-feedback and problem-solving tasks helped in creating trust and influencing change, even in situations where coaches were less senior or less experienced than the birth attendants they coached. However, in some facilities, this age gap remained a challenge. Initial results from pilot studies and data from the larger trial have indicated that the program was able to increase essential birth practices in facilities, even when a coach was not present.^19^^,^^34^

Checklists alone do not resolve all barriers to behavior change.

At the same time, the implementation of the intervention across 60 study facilities and 24 districts (representing a population of approximately 60 million) in the state of Uttar Pradesh as part of the BetterBirth Trial suggests that with the same level of resources, the BetterBirth Program is replicable in a variety of facilities in Uttar Pradesh and in other similar contexts globally. We partially attribute the scale-up to 60 sites to the robust cloud-based data collection and feedback system, Pulse, which proved to be a unique and valuable asset in the process of coaching. However, lower-cost, lower-tech models may also prove to be equally effective in ensuring a robust monitoring and data-feedback loop to facilitate bringing the coaching-based intervention to scale.

Because we implemented the BetterBirth Program in the context of a matched-pair, cluster-randomized controlled trial, we followed a strict trial protocol for the intensity and frequency of coaching. For each of the 60 facilities, the intervention closely adhered to the protocol and the outlined implementation pathway (Engage, Launch, and 43 Support visits). However, outside of a trial context, a truly provider-centered (and therefore adaptive) coaching approach would involve adjusting the timing of coaching visits to match the needs of the facilities and the hours of birth attendants who had less exposure to coaching during daylight hours. Still, several organizations across India are currently testing coaching-centered interventions of various intensities, suggesting that a coaching-based model could be realistic for this context, albeit with fewer facility visits. Jhpiego's SCC implementation across the state of Rajasthan, India, includes facility-based coaching visits every 2 weeks for the first 2 months, followed by 4 once-per-month coaching visits,^35^^,^^36^ and the Technical Support Unit in Uttar Pradesh has implemented monthly coaching visits for a period of 6 months for a childbirth case-sheet.^37^^,^^38^ Given contextual limitations (such as high transfer rates of facility staff across Uttar Pradesh, including CQCs, and the safety concerns and significant distances that prevented the all-female nurse coaches from coaching in facilities after dark), we need more research on how to best structure and customize coaching-centered interventions like the BetterBirth Program in order to achieve and sustain the most effective adoption of essential birth practices.

## CONCLUSION

The BetterBirth Program was centered around coaching in an effort to encourage the consistent, effective delivery of essential birth practices through adoption and use of the SCC, and to sustain this change through individual and facility- or team-level empowerment. The trial showed an improvement in performance of these practices after only 2 months of the intervention.^34^ These results suggest that the BetterBirth strategy of implementing the WHO SCC with coaching can be a method for achieving change in facility-based childbirth care.^34^ However, further research is needed to clarify which aspects of coaching-centered interventions contribute most to increasing use and sustainability of the SCC and to consistent adoption of essential birth practices. Other coaching-based interventions using the SCC have incorporated technical skills training in addition to coaching.^35^^,^^37^^,^^39^ For example, Jhpiego incorporated a 1.5-day clinical training,^35^ and the Technical Support Unit created a 3-day technical training.^37^^,^^38^ Additional research is needed to understand which components of these coaching-based interventions influence sustainable behavior change and consistent application of essential birth practices using the SCC. Should health systems choose to integrate such a strategy to improve quality of care, understanding how it should be integrated into existing supervision models will be important. The goal of the BetterBirth Trial was to test effectiveness of a coaching-based approach to improve quality of care. Therefore, a highly structured, intensive intervention protocol was developed, based on the best available evidence on intensity and duration of coaching. Some elements to enhance sustainability were incorporated, such as the CQC, but overall, BetterBirth was designed to test effectiveness rather than sustainability. Therefore, there is ample scope for additional programmatic innovation to develop more sustainable models. As this becomes a public model for improving facility-based quality of care, sustaining funding for coaching visits and understanding how to prioritize among facilities appropriately will be important factors for sustainability and feasibility.

## Supplementary Material

Supplement

## Notes

**Peer Reviewed**
