# Supplementary material for: The BetterBirth Program: Pursuing Effective Adoption and Sustained Use of the WHO Safe Childbirth Checklist Through Coaching-Based Implementation in Uttar Pradesh, India
Source: Glob Health Sci Pract. 2017 Jun 27;5(2):232–43. doi: 10.9745/GHSP-D-16-00411 (PMC5487086; doi:10.9745/GHSP-D-16-00411)
Supplement: Supplement [file GHSP-D-16-00411_index.html]

Supplement to The BetterBirth Program: Pursuing Effective Adoption and Sustained Use of the WHO Safe Childbirth Checklist Through Coaching-Based Implementation in Uttar Pradesh, India | Global Health: Science and Practice

## Supplement

**Files in this Data Supplement:**

- Safe Childbirth Checklist - Text s01, PDF
